# Tubal Stump Ectopic Pregnancy Following Salpingectomy: A Rare Diagnostic Challenge

**DOI:** 10.7759/cureus.92869

**Published:** 2025-09-21

**Authors:** Madison Sacks, Steven Lewis

**Affiliations:** 1 Obstetrics and Gynecology, Edward Via College of Osteopathic Medicine, Spartanburg, USA

**Keywords:** complete salpingectomy, fallopian tube damage, fallopian tube surgery, fertility sparing surgery, laparoscopic ectopic, recurrent ectopic, recurrent ectopic pregnancy, ruptured ectopic pregnancy, tubal stump ectopic, women fertility

## Abstract

A tubal stump ectopic pregnancy is a rare but serious complication following salpingectomy. This case report not only highlights the rarity of this event but also the potential diagnostic challenges involved.

A 29-year-old woman with a history of ectopic pregnancy and prior left salpingectomy presented to the emergency department with abdominal pain and bleeding during early pregnancy. She reported bilateral lower abdominal pain, which had progressively worsened over two days. Her pregnancy had been recently confirmed, and her obstetrician-gynecologist (OBGYN) was monitoring her beta-human chorionic gonadotropin (HCG) levels. At the time of presentation to the emergency room, her beta-HCG levels had decreased compared to the levels recorded a week earlier. Ultrasound evaluation revealed a right ectopic pregnancy with possible hemoperitoneum. Laparoscopic evaluation subsequently identified the ectopic pregnancy in the left fallopian tube stump. The patient’s hemoglobin level at presentation was 7.4 g/dL, necessitating a blood transfusion. The patient underwent successful surgical intervention and was discharged the following day.

There is limited literature regarding tubal stump ectopic pregnancies, and no established guidelines exist to address their prevention. One case study has suggested a potential association between tubal stump length and the risk of future tubal stump ectopic pregnancy. Maintaining a high index of suspicion is critical in such cases, especially when clinical symptoms are non-specific and may mimic other gynecologic conditions. The patient’s preservation of fertility is a testament to the success of early recognition and surgical intervention in such complex cases. The initial misinterpretation of ultrasound findings suggests the need for advanced imaging techniques to confirm the precise location of rare ectopic pregnancies. Further exploration of this area could help improve diagnostic accuracy and patient outcomes.

## Introduction

A tubal stump ectopic pregnancy, a particularly rare occurrence, happens when the blastocyst of an early embryo implants in a small remnant of the fallopian tube following a salpingectomy [1]. In a prior study, tubal stump ectopic pregnancies were found to have an incidence of 0.4% among the 1,466 ectopic pregnancies [2].

Ectopic pregnancies are a critical complication of pregnancy that must not be overlooked, as they carry a significant risk of morbidity and mortality. They account for 5%-10% of all pregnancy-related deaths, despite advancements in their management [3]. Physicians should maintain a high index of suspicion when a woman presents with symptoms such as vaginal bleeding and/or abdominal pain, particularly if she has risk factors for ectopic pregnancy. Common risk factors include a history of pelvic inflammatory disease (PID), previous ectopic pregnancies, abdominal surgery or trauma, smoking, endometriosis, and the use of intrauterine devices (IUDs) if pregnancy occurs [4]. The non-specific symptoms of ectopic pregnancies often mimic those of other conditions, complicating diagnosis. Currently, the standard diagnostic approach involves ultrasound and measurement of beta-human chorionic gonadotropin (HCG) levels [5].

By presenting this unique case, we aim to enhance the understanding of tubal stump ectopic pregnancies and their clinical implications.

## Case presentation

On March 5, 2024, a 29-year-old female (G3P0020) was referred to the emergency department from her obstetrician-gynecologist (OBGYN) for evaluation of a suspected ruptured ectopic pregnancy. The patient had a significant medical history, including two previous ectopic pregnancies and a left salpingectomy performed in 2023.

The patient presented with bilateral lower abdominal pain that had progressively worsened over the past two days, accompanied by vaginal bleeding and nausea. Recently, she had confirmed her pregnancy, and her OBGYN had been monitoring her beta-HCG levels, which had declined from 3,775 mIU/mL on February 28 to 2,336 mIU/mL at the time of her emergency department visit. The outpatient evaluation revealed concerning signs consistent with a ruptured ectopic pregnancy, with ultrasound findings indicating hemoperitoneum. Ultrasound showed a small volume of anechoic free fluid in the dependent cul-de-sac only.

Upon examination in the emergency department, the patient appeared non-toxic but exhibited marked tenderness in the bilateral lower quadrants with rebound tenderness; her skin was warm and dry. Vital signs upon admission were as follows: blood pressure of 105/73 mmHg, pulse of 96 bpm, and temperature of 100.8°F. The patient’s shock index (SI) was 0.91, consistent with early hemodynamic instability. In the setting of suspected intra-abdominal bleeding, this elevated SI is a red flag for compensated shock.

A bedside point-of-care pelvic ultrasound, conducted by the emergency department physician, revealed a significant volume of fluid in the abdomen and pelvis, along with a large right adnexal mass, raising suspicion for a ruptured ectopic pregnancy with hemoperitoneum. The gestational sac appeared to be located outside the uterus on the right side. Laboratory results indicated a hemoglobin level of 7.4 g/dL, slight hypokalemia at 3.1 mmol/L, and normal coagulation parameters. Notably, her hemoglobin had been 12 g/dL two days prior.

The patient received 1,000 mg of acetaminophen and a 50 mcg fentanyl injection for pain management before being transferred to a surgical facility for intervention. Upon arrival at the new hospital, she was administered a 5-325 mg hydrocodone-acetaminophen tablet and a 500 mg metronidazole tablet for bacterial vaginosis. Examination findings were consistent with the previous evaluation. Her vital signs upon transfer were as follows: blood pressure of 112/63 mmHg, pulse of 86 bpm, and temperature of 98.6°F.

**Figure 1:**
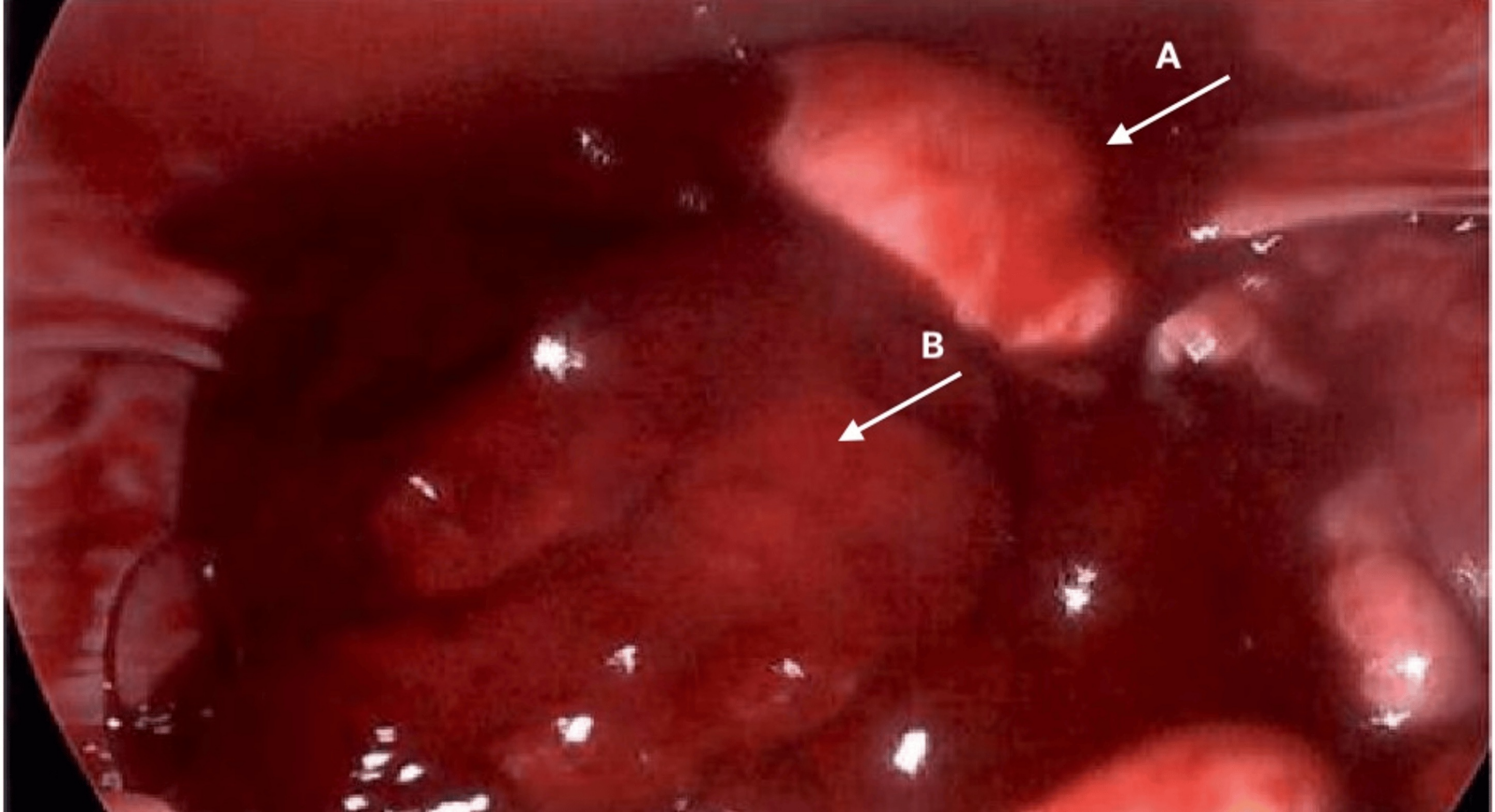
Intraoperative image showing hemoperitoneum A: uterus, B: hemoperitoneum

**Figure 2:**
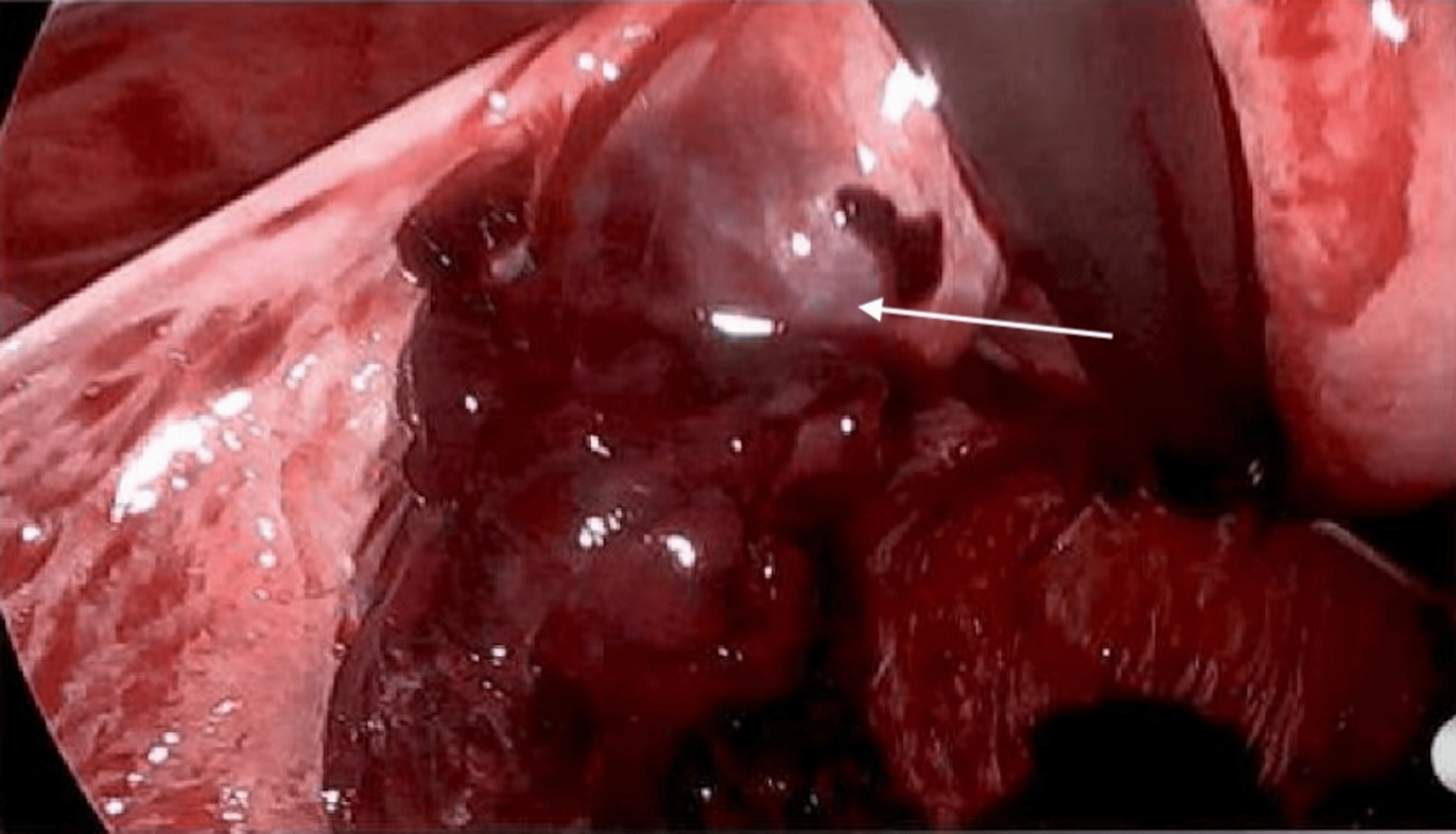
Intraoperative image showing left tubal stump ectopic pregnancy Arrow: ectopic pregnancy

**Figure 3:**
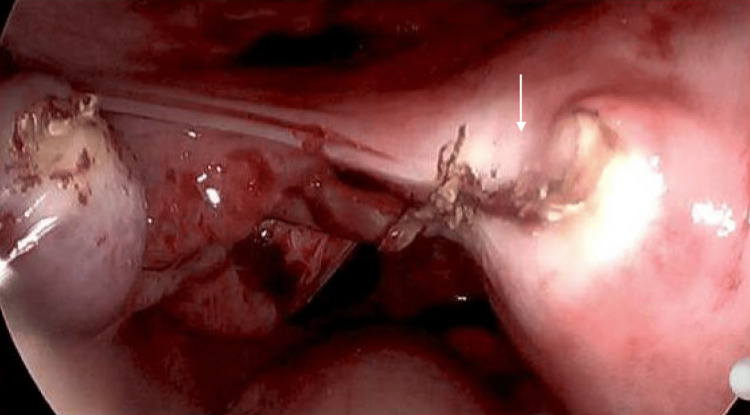
Intraoperative images show post-resection of the ectopic pregnancy Arrow: site of ectopic pregnancy post-removal (Ligasure)

**Figure 4:**
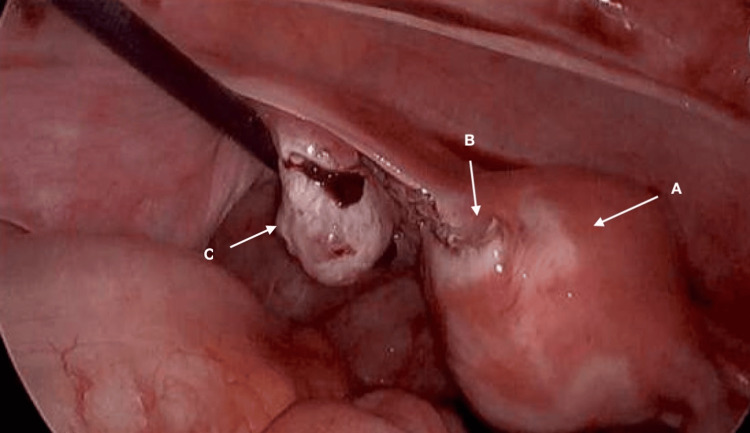
Intraoperative images show post-resection of the ectopic pregnancy with a healthy ovary A: uterus, B: cornu, C: left ovary

She was taken for emergency laparoscopy, during which two units of packed red blood cells were transfused, raising her hemoglobin to 10 g/dL. Operative findings revealed 1,000 mL of blood in the abdominal cavity, which was aspirated. Contrary to initial imaging that suggested a right adnexal mass, surgical exploration identified a ruptured ectopic pregnancy (Figure 1) located in the proximal stump of the left fallopian tube, approximately 2 cm in diameter (Figure 2). The right fallopian tube was found to be healthy, and the uterine cornu was intact. A bipolar energy sealer/divider device (Ligasure) was utilized to transect the proximal fallopian tubal stump immediately adjacent to the uterine corpus (Figures 3, 4). Pathology report of the specimen showed chorionic villi and a blood clot consistent with ectopic pregnancy.

The following day, the patient was ambulating without difficulty, pain was well controlled, and she was ready for discharge, with instructions to follow up with her outpatient OBGYN in one week. The patient’s postoperative hemoglobin was 10.4 g/dL after transfusions and concurrent fluid resuscitation. The patient expressed relief that the ectopic pregnancy was on the left side, preserving her right fallopian tube and maintaining her fertility potential. In March 2025, she went on to have a successful vaginal birth.

## Discussion

A definitive diagnosis of an ectopic pregnancy is made by the visualization of a gestational sac located outside the uterus, most commonly in the adnexa. While the ampulla of the fallopian tube is the most frequent site for an ectopic pregnancy, they can, although rarely, occur in the interstitium, cornua, cervix, ovaries, and peritoneum [5].

Ectopic pregnancies account for approximately 2% of all pregnancies and are a leading cause of maternal morbidity and mortality, with ruptured ectopic pregnancies posing a significant emergency in gynecologic practice [6]. The management of ectopic pregnancies has evolved, with a shift toward medical management using agents such as methotrexate in certain cases. However, this case emphasizes the continued necessity for surgical intervention, particularly in high-risk situations like significant hemoperitoneum and worsening beta-HCG levels, as in our patient. The approach to surgical management depends on factors such as blood loss, the patient’s physical health, and underlying comorbidities. In this case, timely diagnosis and the use of beta-HCG levels in conjunction with ultrasound were critical to confirm the ectopic pregnancy and facilitate early intervention.

Several studies have documented the association of prior ectopic pregnancies and salpingectomy with an increased risk of future ectopic pregnancies, as seen in our case. The recurrence rate of ectopic pregnancy in women with a history of a previous ectopic pregnancy is 10%-20% [7]. This highlights the necessity for close monitoring of beta-HCG levels in such patients and early imaging to avoid life-threatening complications, such as rupture. Moreover, this case reinforces the importance of maintaining a high index of suspicion, particularly when clinical symptoms are non-specific and can mimic other gynecologic conditions. Vigilance in recognizing ectopic pregnancy in women with risk factors, such as a previous ectopic pregnancy or salpingectomy, is essential for early diagnosis and effective management.

The patient’s favorable outcome, with preservation of fertility due to the intact right fallopian tube, is a testament to the success of early recognition and surgical intervention in such complex cases. This was further supported by her emotional relief upon learning the left fallopian tube was affected, providing insight into the psychological impact of preserving fertility in women with a history of ectopic pregnancies.

One limitation of this case is the initial misinterpretation of the ultrasound findings, which suggested a right adnexal mass when the ectopic pregnancy was ultimately located in the left fallopian tube stump. This underscores the limitations of ultrasound in diagnosing ectopic pregnancies and highlights the potential benefit of advanced imaging techniques in confirming the location of rare ectopic pregnancies. More advanced imaging techniques, such as magnetic resonance imaging (MRI), could potentially provide more accurate localization of ectopic pregnancies [8]. The factor of human error must also not be ignored, as assumptions based on a patient’s prior medical history may lead to misdiagnosis or a delay in diagnosis.

The literature search did not yield any specific recommendations or guidelines regarding the optimal amount of tube tissue to leave behind during a salpingectomy to prevent recurrence. The general practice is to leave as little of the tube as possible. One study suggested that the remaining intact tubal lamina could be a possible risk factor for the recurrence of ectopic pregnancy in the tubal stump. Therefore, they proposed the use of hysterosalpingography after adequate use of bipolar energy sealer/divider devices for the transection of the tube to assess tubal patency [9], enabling further interventions if tubal patency is preserved. On the other hand, another case study suggests that the recurrence rate is independent of tubal length and recommends leaving a longer stump to minimize bleeding risk and facilitate easier surgical procedures [10].

## Conclusions

This case emphasizes the importance of early recognition, careful monitoring, and timely intervention in patients with a history of ectopic pregnancies. It adds to the body of literature regarding the recurrence of ectopic pregnancies and highlights the need for ongoing research into advanced diagnostic techniques and the long-term fertility outcomes of women with a history of ectopic pregnancy. Further research is also needed to determine whether post-salpingectomy tubal length influences the recurrence of stump ectopic pregnancies.
